# Supporting the Transition to Pediatric Residency: A Transitional Elective Block for International Medical Graduates

**DOI:** 10.7759/cureus.112812

**Published:** 2026-07-16

**Authors:** Osama Aloudat, Amjad Abdelrahim, Luiza Seleguim, Cansu Tokat, Kayla Heller

**Affiliations:** 1 Pediatrics, SSM Health Cardinal Glennon Children’s Hospital, Saint Louis University School of Medicine, St. Louis, USA

**Keywords:** international medical graduates, medical education, onboarding, pediatric residency, residency transition

## Abstract

Background

International Medical Graduates (IMGs) entering U.S. residency programs often encounter challenges during the transition to internship, including unfamiliarity with the U.S. healthcare system, clinical workflows, electronic medical records (EMRs), and expectations within graduate medical education. Despite these challenges, structured onboarding interventions designed to support IMG transition remain limited.

Objective

To describe the implementation of a Transitional Elective Block (TEB) designed to support IMG pediatric interns during the transition to residency and to evaluate its early educational outcomes using quantitative self-reported measures and qualitative participant feedback.

Methods

A four-week transitional elective block was implemented for incoming IMG pediatric interns during the summers of 2024 and 2025 at a university-affiliated pediatric residency program. The curriculum emphasized supervised clinical exposure, mentorship, patient presentations, handoff communication, electronic medical record (EMR) navigation, and clinical workflow acclimation. Program evaluation used a mixed-methods approach, including baseline and follow-up surveys assessing self-reported confidence using five-point Likert scales and open-ended responses. Quantitative analyses were exploratory and performed using nonparametric statistical methods, while qualitative responses were analyzed using rapid content analysis.

Results

At baseline, TEB participants reported lower self-reported confidence across multiple domains than non-participants. By three months of internship, participants demonstrated greater relative improvements in confidence, partially narrowing baseline differences. All TEB participants reported that they would recommend beginning residency with the elective. At follow-up, participants reported higher self-rated clinical skill and comfort across several domains, whereas EMR use and system navigation remained common challenges across the intern cohort. Qualitative responses highlighted improved confidence, a clearer understanding of residency expectations, and the value of structured mentorship and early clinical exposure. Given the pilot study design and small sample size, quantitative findings should be interpreted as exploratory and hypothesis-generating.

Conclusions

The Transitional Elective Block was feasible to implement using existing residency resources and was well accepted by participants. These preliminary findings suggest that a structured transitional elective may enhance perceived preparedness for the transition to residency among international medical graduates. Larger multicenter studies incorporating objective measures of clinical performance are needed to evaluate the effectiveness and generalizability of this educational model.

## Introduction

International medical graduates (IMGs) represent a critical component of the U.S. pediatric workforce and play an important role in addressing physician shortages, particularly in primary care and underserved communities [1-3]. Despite their essential contributions, IMGs frequently encounter distinct challenges during the transition into U.S. residency training, including unfamiliarity with the U.S. healthcare system, institutional workflows, communication expectations, and electronic medical record (EMR) platforms [2,4,5]. Chen et al. [6] identified that non-U.S.-born IMGs face professional challenges, including navigating unfamiliar clinical hierarchies and communication norms, while Symes et al. [7], in a national survey of more than 7,800 J-1 visa IMG residents, reported that cultural differences, navigating the U.S. healthcare system, and separation from family were among the most common wellness concerns. These challenges may contribute to reduced confidence, increased stress, and delayed integration during the early months of residency training [2].

Prior studies have documented disparities in the early residency experiences of IMGs, particularly during postgraduate year one; however, few published interventions have described structured, IMG-specific transition curricula accompanied by outcome data [3-5]. A recent scoping review by Nwankwo et al. identified 29 educational support strategies for IMGs, including communication workshops, orientation programs, and clinical attachments [8]. However, most interventions were conducted in Australia and the United Kingdom, with limited evidence from U.S.-based residency programs [8]. Tsugawa et al. [9] demonstrated, in a large Medicare study, that patients treated by international medical graduates had lower 30-day mortality than those treated by U.S. medical graduates, while Jimenez-Gomez et al. found that IMGs in pediatric residency performed comparably to their U.S. medical graduate peers [10]. Although these findings suggest that IMGs ultimately achieve comparable clinical competency, the initial transition into residency remains a particularly vulnerable period [2,3]. As emphasized by Kehoe et al., organizational support and cultural awareness are essential to facilitate a successful transition for IMG trainees [11].

The Transitional Elective Block (TEB) was developed after program leadership recognized that early performance concerns among some IMG interns often reflected system-level transition barriers rather than individual deficiencies. Initial iterations of the TEB were implemented to support interns experiencing difficulties during their first clinical rotations. Over time, however, the program evolved into a proactive, structured elective designed to facilitate successful integration into residency, enhance early clinical performance, and reduce the need for formal remediation.

## Materials and methods

Study design and setting

This pilot educational innovation was implemented at a tertiary children's hospital, SSM Health Cardinal Glennon Children’s Hospital, St. Louis, Missouri, United States, within a university-affiliated pediatric residency program. The program includes a diverse cohort of international medical graduates (IMGs).

Participants

The Transitional Elective Block (TEB) was offered to incoming IMG pediatric interns during the first quarter of intern year across two cohorts (July 2024 and July 2025). In 2024, 3 of 23 interns (3 of 10 IMGs) participated in the TEB. In 2025, 7 of 22 interns (7 of 17 IMGs) participated. The elective was available to any incoming IMG intern seeking additional support during the transition to residency, and participation was entirely voluntary. Participants were 50% female and represented a diverse range of countries across three continents. The interval between medical school graduation and residency ranged from less than 1 year to more than 5 years.

Intervention: transitional elective block (TEB)

The TEB is a four-week structured rotation designed to provide International Medical Graduates with early, supported exposure to clinical practice and residency expectations within the U.S. healthcare system. The primary goals of the TEB are to enhance interns' confidence, improve familiarity with clinical workflows and institutional systems, and facilitate early integration into the residency learning environment.

The TEB is a proactive onboarding elective designed to accelerate system acclimation and strengthen early clinical communication skills. It combines supervised clinical experiences across inpatient pediatrics, emergency medicine, and ambulatory settings with focused skill development in patient presentations, handoff preparation, and interdisciplinary communication. System-based orientation is integrated throughout the rotation, including structured exposure to electronic medical record (EMR) navigation and institutional workflows. Mentorship and reflection are supported through faculty mentorship meetings and structured debriefing sessions. The curriculum emphasizes graduated responsibility, direct observation, and deliberate practice within a low-stakes learning environment, allowing IMG interns to develop core clinical and communication skills while receiving targeted feedback and support during the early phase of residency training.

Implementation

The TEB was implemented across two intern cohorts (2024-2025) and offered as an elective to incoming IMG interns. Participants rotated through multiple clinical settings, including inpatient pediatric services, the well-baby nursery, the emergency department, and the ambulatory clinic. Based on feedback from the initial cohort, a neonatal intensive care unit (NICU) experience was added in 2025. Protected time was incorporated throughout the rotation for mentorship, direct observation, and targeted skills practice.

Faculty preceptors received the resident schedule along with a brief written summary of the rotation's goals and objectives, emphasizing patient presentations, handoff communication, EMR navigation, and clinical workflow acclimation. Senior residents were similarly oriented to the goals of the rotation, and, whenever feasible, TEB participants were paired with senior residents recognized for strong mentorship skills. Ongoing feedback from participants and faculty was reviewed regularly and used to iteratively refine curricular components, particularly EMR training and patient handoff preparation. The TEB required no additional funding and was implemented using existing faculty supervision, clinical rotations, and scheduled elective time.

Data collection

Program evaluation used a mixed-methods approach, with anonymous baseline and follow-up surveys assessing self-reported confidence using five-point Likert scales and open-ended questions. The survey instrument was developed locally for program evaluation purposes, and no formal evidence of validity has been established.

Statistical analysis

Quantitative analyses were exploratory and hypothesis-generating. Between-group comparisons of Likert-scale responses were performed using the Mann-Whitney U test due to the small sample size and non-normality of the responses. Following the initial analysis, the Benjamini-Hochberg correction was applied to control the false discovery rate associated with multiple comparisons. The primary analysis compared TEB participants with a combined control group consisting of all non-TEB participants, including both IMG and U.S. medical graduate interns. An additional exploratory analysis using the Kruskal-Wallis test compared three groups: TEB participants, non-TEB IMG participants, and non-TEB U.S. medical graduate participants. However, the sample sizes were insufficient to support meaningful statistical conclusions. Because residency classes consist of both IMG and U.S. medical graduate interns who are expected to meet the same performance standards and certification requirements, comparing TEB participants with all non-participants was considered the most appropriate approach for evaluating the overall impact of the elective. Qualitative responses were analyzed using rapid content analysis. Responses were coded into recurrent themes, and discrepancies were resolved through author consensus.

The focus of data collection evolved between the first and second cohorts. During the initial implementation, program evaluation focused primarily on IMG participants to determine whether those who completed the TEB differed from their IMG peers. The primary objectives were to evaluate the perceived value of the elective, assess its impact on the transition to residency, and inform future curricular refinements. In 2025, the evaluation expanded to include all incoming interns, allowing for a broader assessment of the elective's potential impact across the residency program. These data were used to explore whether a structured transitional elective might benefit all interns, including both IMGs and U.S. medical graduates.

Ethical considerations

All data were collected and analyzed anonymously, and no personal identifiers were included. The requirement for informed consent was waived.

## Results

2024 cohort

Seven International Medical Graduates (IMGs) across two intern cohorts completed baseline and follow-up surveys, of whom three (3/7, 43%) participated in the TEB. At baseline, TEB participants reported lower self-reported confidence than IMG non-participants across multiple domains, including familiarity with the U.S. healthcare system, patient presentations during rounds, medical terminology, and the management of complex pediatric patients.

**Figure 1:**
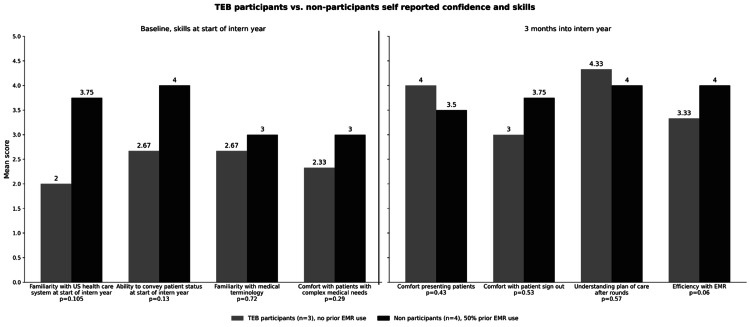
Pre- and post-TEB confidence and preparedness among IMG interns Mean self-reported confidence and preparedness scores among International Medical Graduate (IMG) interns participating in the Transitional Elective Block (TEB) compared with IMG non-participants at baseline and 3-month follow-up. Responses were measured using a 5-point Likert scale. Results are descriptive.

Three months into the internship, TEB participants demonstrated greater relative improvements in self-reported confidence across several domains, including patient presentations, handoff preparedness, and understanding plans of care following rounds, resulting in a partial narrowing of confidence differences between TEB participants and non-participants (Figure 1). All TEB participants (3/3, 100%) reported that they would recommend beginning residency with the Transitional Elective Block. Given the small sample size, formal statistical comparisons were not performed for the 2024 cohort. Qualitative responses highlighted improved clarity in residency expectations, increased confidence during rounds, and reduced stress in the early months of the internship. Persistent challenges related to electronic medical record (EMR) efficiency and patient handoff were also identified.

2025 Cohort

**Figure 2:**
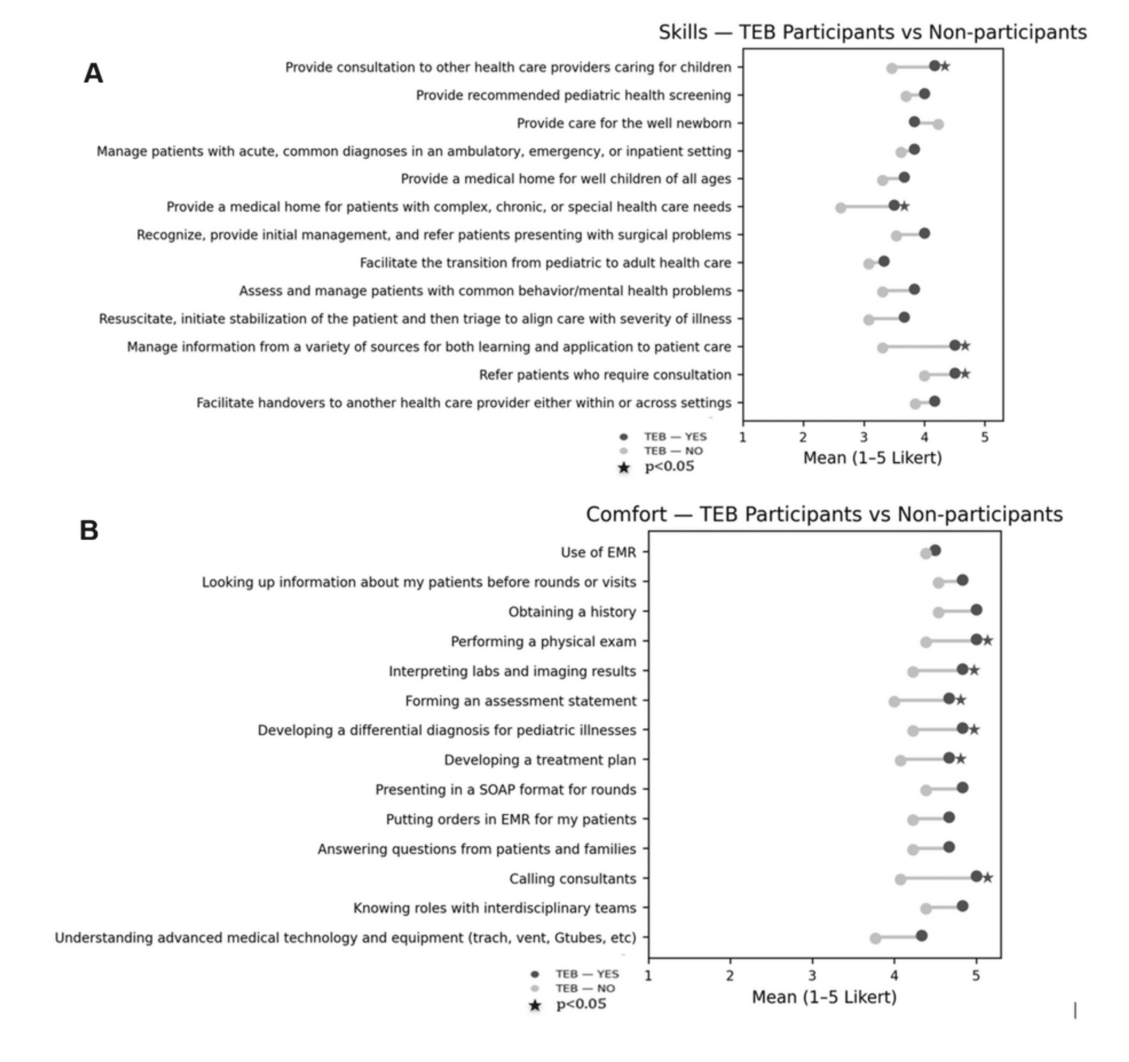
January 2026 follow-up survey results comparing interns who completed the TEB with non-participants (A) Self-reported pediatric skill scores by Transitional Elective Block (TEB) participation; (B) self-reported clinical comfort scores by TEB participation. 2A displays mean self-reported pediatric skill scores across core clinical competencies, including consultation, information management, care of patients with complex needs, and referral skills. 2B displays mean self-reported comfort scores with core intern clinical tasks, including physical examination, clinical reasoning, treatment planning, communication, and electronic medical record use. Bars represent mean responses on a 5-point Likert scale. Light bars represent interns who completed the TEB, whereas dark bars represent non-participants. Error bars indicate standard deviation. Asterisks (*) indicate exploratory statistical significance (p < 0.05).

All incoming interns in 2025 were invited to complete a baseline survey. Of the 22 interns, 17 were IMGs, and 7 elected to participate in the TEB (32% of all interns and 41% of IMGs). Eighteen interns completed the baseline survey. In January 2026, 19 interns completed the follow-up survey, including 15 IMGs and 4 U.S. medical graduates. Six respondents reported completing a transitional elective during the first six months of internship and completed additional questions regarding their experience with the TEB. At follow-up, TEB participants (n = 6) demonstrated higher self-reported pediatric skill and clinical comfort scores across multiple domains than non-participants (n = 13), which included both IMGs and U.S. medical graduates (Figures 2A, 2B).

Table 1 summarizes self-reported pediatric skill scores by TEB participation. Participants demonstrated higher mean scores across several domains, including consultation skills, information management, and referral practices, with statistically significant differences observed for selected items. Table 2 presents self-reported clinical comfort scores, demonstrating higher comfort levels among TEB participants in multiple areas, including physical examination, clinical reasoning, treatment plan development, and consultant communication.

**Table 1:** Self-reported pediatric skills by transitional elective block participation TEB, Transitional Elective Block; EMR, electronic medical record; MWU, Mann-Whitney U test; FDR, false discovery rate. A post hoc Benjamini-Hochberg FDR adjustment was applied for multiple comparisons.

Item	TEB Participant Mean	TEB Non-Participant Mean	Effect Size (r)	Mann-Whitney U p	FDR-adjusted p
Provide consultation to other healthcare providers caring for children	4.17	3.46	-0.55	0.031	0.12
Provide recommended pediatric health screening	4.00	3.69	-0.26	0.306	0.34
Provide care for the well newborn	3.83	4.23	0.28	0.299	0.34
Manage patients with acute, common diagnoses	3.83	3.62	-0.15	0.531	0.55
Provide a medical home for well children of all ages	3.67	3.31	-0.33	0.222	0.30
Provide a medical home for patients with complex, chronic, or special healthcare needs	3.50	2.62	-0.58	0.041	0.12
Recognize, initially manage, and refer patients with surgical problems	4.00	3.54	-0.39	0.144	0.23
Facilitate transition from pediatric to adult healthcare	3.33	3.08	-0.19	0.486	0.52
Assess and manage common behavioral or mental health conditions	3.83	3.31	-0.35	0.223	0.30
Resuscitate, stabilize, and appropriately triage critically ill patients	3.67	3.08	-0.41	0.134	0.23
Manage information from multiple sources for learning and patient care	4.50	3.31	-0.73	0.007	0.09
Refer patients requiring specialty consultation	4.50	4.00	-0.46	0.046	0.12
Facilitate patient handoffs across providers and settings	4.17	3.85	-0.28	0.233	0.30

**Table 2:** Self-reported clinical comfort by transitional elective block participation TEB, Transitional Elective Block; EMR, electronic medical record; MWU, Mann-Whitney U test; FDR, false discovery rate. A post hoc Benjamini-Hochberg FDR adjustment was applied for multiple comparisons.

Item	TEB Participant Mean	TEB Non-Participant Mean	Effect Size (r)	Mann-Whitney U p	FDR-adjusted p
Use of the electronic medical record (EMR)	4.50	4.38	-0.08	0.805	0.81
Looking up patient information before rounds or clinic	4.83	4.54	-0.30	0.249	0.31
Obtaining a history	5.00	4.54	-0.46	0.057	0.14
Performing a physical examination	5.00	4.38	-0.54	0.034	0.12
Interpreting laboratory and imaging results	4.83	4.23	-0.54	0.043	0.12
Formulating an assessment	4.67	4.00	-0.56	0.032	0.12
Developing a differential diagnosis	4.83	4.23	-0.60	0.018	0.12
Developing a treatment plan	4.67	4.08	-0.59	0.010	0.09
Presenting patients using SOAP format	4.83	4.38	-0.45	0.085	0.18
Entering orders in the EMR	4.67	4.23	-0.39	0.150	0.23
Answering questions from patients and families	4.67	4.23	-0.39	0.150	0.23
Calling consultants	5.00	4.08	-0.69	0.010	0.09
Understanding interdisciplinary team roles	4.83	4.38	-0.45	0.085	0.18
Understanding advanced medical technology (tracheostomy, ventilators, gastrostomy tubes, etc.)	4.33	3.77	-0.41	0.098	0.19

Exploratory analyses identified statistically significant differences favoring TEB participants in selected skill domains, including providing consultations to other healthcare professionals, managing information from multiple sources for learning and patient care, and appropriately referring patients for specialty consultation. Although several additional comparisons did not reach statistical significance, the observed effect sizes suggest these findings warrant further investigation in larger cohorts. Given the limited sample size and multiple item-level comparisons, these results should be interpreted cautiously and considered hypothesis-generating.

By mid-intern year, self-reported confidence and clinical comfort were generally high across the overall intern cohort, suggesting convergence in perceived preparedness over time. However, EMR use and institutional system navigation remained among the most commonly reported challenges, regardless of training background. Open-ended responses consistently identified EMR navigation, familiarity with institutional systems, and adaptation to clinical workflow expectations as ongoing challenges. Participants frequently cited support from senior residents and attending physicians as key facilitators of a successful transition to residency. Additional facilitators included peer support, structured intern orientation programs, and IMG-focused initiatives such as transitional electives and preparatory courses.

## Discussion

This pilot educational innovation suggests that a structured Transitional Elective Block (TEB) may support International Medical Graduate (IMG) interns during the early transition to residency. Although TEB participants entered residency with lower self-reported confidence than their peers, they demonstrated greater relative improvements over time, suggesting that structured early support may help mitigate challenges encountered during the transition into U.S. residency training.

The TEB addresses an important gap in residency onboarding by providing early system orientation, communication skills training, and mentorship within a supportive learning environment. Participants reported high satisfaction with the elective and consistently recommended the experience to future interns. The core components of the TEB-including supervised clinical exposure, communication skills practice, and system orientation-were implemented using existing residency resources and may be adaptable across residency programs of varying size and structure. This approach aligns with previously described intern boot camp models that have demonstrated improvements in learner confidence and clinical preparedness. Cohen et al. [12] reported that simulation-based mastery learning significantly improved intern performance across assessed skills, while Devon et al. [13] demonstrated sustained improvements in self-efficacy following a pediatric boot camp. However, Rideout et al. found no significant improvement in milestone-based performance among boot camp participants, highlighting the importance of evaluating educational interventions using objective outcomes in addition to self-reported measures [14]. Future studies should therefore evaluate whether participation in the TEB is associated with improvements in milestone achievement, entrustable professional activities, faculty evaluations, and other objective measures of clinical performance.

Despite the limitations of a small sample size and reliance on self-reported outcomes, our findings are consistent with previous literature demonstrating that IMGs and U.S. medical graduates achieve comparable competency by graduation while recognizing that the early transition period remains particularly challenging for many IMG trainees [9-11]. Songara et al. demonstrated comparable milestone progression between IMGs and U.S. medical graduates in family medicine, with improved performance observed in residency programs with greater IMG representation, suggesting that institutional familiarity with IMG integration may facilitate trainee success [15]. By targeting this vulnerable transition period, the TEB reframes early performance variability as a systems-level onboarding challenge rather than an individual deficiency, potentially reducing the need for formal remediation later in training. This perspective is consistent with acculturation theory. Al-Haddad emphasized that IMG performance should be understood within the broader context of migration and acculturation and that successful transition interventions should address organizational, social, and personal dimensions of the adaptation process [16]. By normalizing structured support for IMG trainees, the TEB may foster a more supportive learning environment, promote successful integration into residency, and potentially improve long-term trainee well-being.

Using Kirkpatrick's model of evaluation, the current findings primarily support improvements in learner experience and perceived preparedness (Levels 1 and 2) [17]. Although this pilot study did not evaluate behavioral change, patient outcomes, or long-term performance measures (Levels 3 and 4), participants and program leadership perceived the intervention as valuable. Since the implementation of the TEB, faculty awareness of IMG transition challenges has increased, accompanied by greater emphasis on early coaching and expectation-setting during internship. The institution has also developed faculty development sessions addressing topics relevant to IMG transition, including implicit bias and cultural humility. Future evaluation of the TEB will include longitudinal follow-up to assess sustainability, scalability, and objective educational outcomes. As Allen et al. emphasized, outcome-based evaluation should be complemented by approaches that examine how and why educational interventions achieve their effects, representing an important direction for future evaluation of the TEB [18].

Lessons learned

Several important lessons emerged during implementation. Early protected time dedicated to system orientation, communication skills practice, and direct observation appeared to be valuable components of the curriculum. Equally important was maintaining flexibility to refine the curriculum based on participant feedback. Electronic medical record (EMR) efficiency and patient handoff preparation remained persistent challenges across both cohorts and continue to represent important targets for ongoing curricular refinement. These lessons have informed iterative improvements to the TEB and will guide future implementation.

Limitations

This study has several limitations. First, it was conducted within a single residency program with a relatively small sample size, limiting statistical power and generalizability. As a pilot educational innovation, the study was not designed or powered to detect statistically significant differences between groups or establish intervention effectiveness. Although inclusion of two intern cohorts modestly increased the sample size, the curriculum and survey strategy evolved over time, introducing additional heterogeneity. Furthermore, the study did not employ a true pre-post longitudinal design, as the baseline survey focused primarily on IMG participants, whereas the follow-up survey included the broader intern cohort. Consequently, direct longitudinal comparisons should be interpreted with caution.

Quantitative analyses were exploratory. Following post hoc adjustment for multiple comparisons using the Benjamini-Hochberg false discovery rate (FDR) procedure, no comparisons remained statistically significant. Accordingly, these findings should be interpreted as exploratory and hypothesis-generating rather than confirmatory. Outcome measures were limited to self-reported confidence, perceived preparedness, and clinical comfort and did not include objective assessments such as faculty evaluations, entrustable professional activities, milestone performance, Objective Structured Clinical Examinations (OSCEs), validated assessment tools, or patient outcomes.

Participation in the TEB was voluntary, introducing the potential for selection bias. In addition, the primary comparison group included both International Medical Graduates and U.S. medical graduates, whose differing levels of familiarity with the U.S. healthcare system may have introduced confounding. Although this approach reflects the actual residency training environment, these comparisons should be interpreted with caution. Callahan et al. demonstrated that volunteer bias in medical education research may overestimate intervention effects because learners who voluntarily participate in educational programs often demonstrate greater motivation and stronger longitudinal performance [19]. Notably, several TEB participants also pursued additional preparatory experiences before residency, suggesting that learner motivation and self-awareness may have influenced both participation and perceived benefit.

## Conclusions

The Transitional Elective Block was feasible to implement using existing residency resources and was well accepted by participants. These preliminary findings suggest that a structured transitional elective may improve perceived preparedness during the transition to residency, particularly for international medical graduates. Larger, multicenter studies incorporating objective measures of clinical performance and longitudinal follow-up are needed to determine the effectiveness, generalizability, and long-term impact of this educational model.
